# Multifunctional Cement Composites Strain and Damage Sensors Applied on Reinforced Concrete (RC) Structural Elements

**DOI:** 10.3390/ma6030841

**Published:** 2013-03-06

**Authors:** Francisco Javier Baeza, Oscar Galao, Emilio Zornoza, Pedro Garcés

**Affiliations:** Civil Engineering Department, Universidad de Alicante, Ctra. San Vicente s/n, San Vicente del Raspeig 03690, Spain; E-Mails: fj.baeza@ua.es (F.J.B.); oscar.galao@ua.es (O.G.); emilio.zornoza@ua.es (E.Z.)

**Keywords:** strain sensing, cement composite, carbon nanofibers, CNF, carbon fiber, damage sensing

## Abstract

In this research, strain-sensing and damage-sensing functional properties of cement composites have been studied on a conventional reinforced concrete (RC) beam. Carbon nanofiber (CNFCC) and fiber (CFCC) cement composites were used as sensors on a 4 m long RC beam. Different casting conditions (*in situ* or attached), service location (under tension or compression) and electrical contacts (embedded or superficial) were compared. Both CNFCC and CFCC were suitable as strain sensors in reversible (elastic) sensing condition testing. CNFCC showed higher sensitivities (gage factor up to 191.8), while CFCC only reached gage factors values of 178.9 (tension) or 49.5 (compression). Furthermore, damage-sensing tests were run, increasing the applied load progressively up to the RC beam failure. In these conditions, CNFCC sensors were also strain sensitive, but no damage sensing mechanism was detected for the strain levels achieved during the tests. Hence, these cement composites could act as strain sensors, even for severe damaged structures near to their collapse.

## 1. Introduction

Multifunctional cement composites, *i.e.*, cement-based materials reinforced with randomly dispersed discontinuous carbon fibers or carbon nanofibers, are technologically interesting, due to a wide range of functional applications [1,2,3,4,5,6,7,8,9,10,11,12,13,14,15,16,17,18,19,20,21,22,23,24,25,26,27], combined with their good structural behavior and durability [28,29,30,31,32,33,34,35,36,37,38,39]. Amongst these improved mechanical properties, a higher flexural toughness and strength and tensile ductility and strength [28,29,30,31,32,33,36,37] can be quoted. The enhancement of durability is shown as a low drying shrinkage [36] and a better structural behavior under corrosive environment [28,32,33,34]. The exceptional functional properties are directly related to the high electrical conductivity that this type of materials shows. They include different applications as electromagnetic interference shielding material [5,6,7,10] or strain sensors [1,9,10,11,12,13], due to the piezoresistivity (change of the electrical resistivity with strain). The high conductivity is also attractive for other applications, such as electrical contact for several purposes (chloride extraction [2,3,4], cathodic protection [20,21]) or resistance heating (*i.e.*, for deicing [15,22]).

A material’s strain sensing (or self-sensing) capacity can be defined as the response on the volumetric electrical resistivity (proportional and reversible) due to its strain state [1,9,10,11]. If a longitudinal compressive stress is applied, the electrical resistance on that direction is reduced. However, if the material is upon tension, the effect will be the contrary, *i.e.*, an increase on the resistance will be registered. Both effects are reversible in the material’s elastic range; therefore, the electrical resistance returns to its initial value once the load is removed. This application of cement composites is interesting for structural service state monitoring, room occupancy control or vehicle weighing. Nevertheless, damage sensing mechanism is related to the material’s plastic behavior and can be seen as irreversible changes in the electrical resistivity. The sensitivity of the composites will be measured using the gage factor (*GF*), which can be defined as the fractional change of the electrical resistance per strain unit. This parameter can be calculated according to Equation (1), where Δ*R*: change of electrical resistance; *R*_0_: initial electrical resistance; Δ*l*: deformation; *l*_0_: initial length; and *ε*: longitudinal strain:

(1) GF=Δρρ0Δll0=ΔRR0ε

Carbon fiber reinforced cement composite (CFRCC) functional properties have been vastly studied in laboratory conditions and small samples, as above-mentioned, *i.e.*, self-sensing [1,7,8,9,10,11], damage-sensing [9,10,11,19]. Even the addition of nano-admixtures is currently being characterized, and in the last few years, it has indeed been focused on CNT [10,23,24,25,26,27]. However, very few studies have tried to test real scale structural elements. Some of the real applications that have actually been reported include CFRCC as deicing pavement in a highway bridge [22] or as an anode in the electrochemical chloride extraction technique (ECE) of building pillars contaminated with chloride ions [3]. Also, two studies related to damage or strain sensing can be found as traffic monitoring [25] or wireless and embedded CNT networks for damage detection in concrete structures [26].

In the current paper, an application of multifunctional cement composites as strain and damage sensors on RC beams is reported. A comparison between CNF and CF as conductive admixtures in the composites has been made.

## 2. Experimental Program and Materials

**Figure 1:**
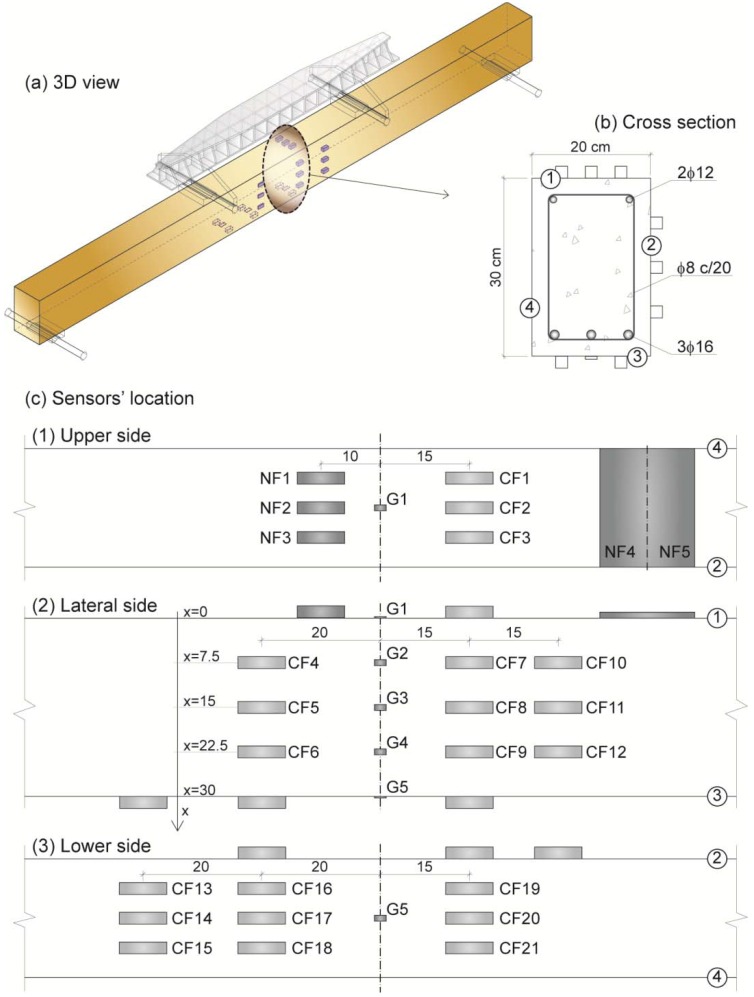
(**a**) Three dimensional (3D) view of the sensors’ distribution; also, loading and support conditions are represented; (**b**) Cross section dimensions and steel rebar’s arrangement; (**c**) Sensors’ location and nomenclature (NF = carbon nanofiber; CF = carbon fiber; G = strain gage).

One RC beam of 0.2 × 0.3 × 3.9 m³ was casted, and 28 multifunctional cement composite sensors were prepared. In Figure 1, the main characteristic of both, beam and sensors, are represented. Figure 1c shows the position of each sensor with respect to the middle cross section of the beam. 

Two different types of test were made, depending on the magnitude of the maximum strains or stresses in the reinforced concrete. Hence, strain-sensing and damage-sensing experimental setups should be distinguished. In the former, once the cracking bending moment has been exceeded, the RC beams behavior can be considered elastic, whereas in the latter, either steel or concrete elements suffer additional and irreversible damage.

### 2.1. Sensor’s Preparation 

Portland cement pastes with CF and CNF additions were fabricated. Portland cement, type CEM I 52.5 R, according to UNE-EN 197-1:2000, was used. Distilled water was used, and the water/cement ratio (w/c) was fixed at 0.5 for all dosages. Five different dosages of conductive admixtures were prepared. The first one was a 2% CNF (by cement mass) cement paste; CNF properties (supplied by Grupo Antolín S.A.) are included in Table 1. The other four dosages were 1% CF (by mass of cement) and used two different fibers Zoltek PANEX35 (CF3) and Hexcel HEXTOW AS4 (CF10), both as received and after being treated superficially in an oxidation process (CF3ox and CF10ox, respectively). CF properties can be seen in Table 2.

**Table 1:** Carbon nanofiber (CNF)—Grupo Antolín type GANF4 properties.

Property	CNF
Fiber diameter (TEM)	20–80 nm
Fiber length (SEM)	>30 µm
Bulk density	>1.97 g/cm^3^
Apparent density	0.060 g/cm^3^
Surface energy	≈100 mJ/m^2^
Specific surface area BET (N_2_)	150–200 m^2^/g
Graphitization degree	≈70%
Resistivity	1 × 10^−3^ Ω m
Metallic particles content	6%–8%

**Table 2:** Carbon fiber (CF) properties.

Property	CF3	CF10
Fiber type	PANEX 35	HEXTOW AS4
Diameter	7.2 µm	7.1 µm
Length	3.5 mm	≈10 mm
Carbon content	95%	94%
Tensile strength	3800 MPa	4480 MPa
Elastic modulus	242 GPa	231 GPa
Resistivity	1.52 × 10^−3^ Ω cm	1.52 × 10^−3^ Ω cm
Density	1.81 g/cm^3^	1.79 g/cm^3^

In order to achieve a uniform dispersion of the conductive admixtures in the mix, specific treatments were used. CNF were previously dispersed in the mix water. A double treatment was applied for this purpose based on prior work on polymeric matrices [38]. First of all, CNF were added to the water and mechanically stirred in an automatic mixer. Afterwards, an ultrasounds treatment was applied to the mix using an ultrasonic device, model Hielschier UP200S. 

CF were subjected also to two types of treatment prior to incorporation in the mix: oxidation and sonication. The first treatment, oxidation, was conducted by placing the fibers in air at 400 °C with a flow of 10 mL/min for 4 h [31]. This treatment served to (i) remove the fiber sizing so as to expose the carbon in the fiber and (ii) to form oxygen-containing functional groups on the surface of the carbon in the fiber, so improving the wettability of the fiber by water and strengthening the fiber-matrix bonding [31]. Subsequent to oxidation, a second treatment was applied on the same fibers. This second treatment, just as for the CNF, involved sonication for 5 min after the fibers had been dispersed in water.

Afterwards, all components (CNF or CF dispersed in water, cement and plasticizer) were poured into an automatic mixer for 5 min. Two different sizes of prismatic samples were prepared: 2 × 2 × 8 cm^3^ and 20 × 0.7 × 8 cm^3^. Another variable of study was the casting method. Some samples were prepared in external molds and, after curing for 28 days immersed in water, were attached to the RC beam using an epoxy resin, Pattex Nural 27. Only a thin layer of resin was applied in order to guarantee the strain transmission between sensors and the beam. Other samples were casted directly upon the RC, *in situ*, after treating it superficially (cleaning plus bonding agent, Sikadur-32 Fix). The final combination of dosage, dimension and location for each particular sensor has been included in Table 3. 

**Table 3:** Sensors properties (dimensions, casting method, location and electrical contacts).

Sensor	Conductive admixture	Casting method	Width (cm)	Thickness (cm)	Position (*x*), Depth (cm)
NF1	2% CNF	Attached	2	2	0
NF2	2% CNF	Attached	2	2	0
NF3	2% CNF	Attached	2	2	0
NF4	2% CNF	*In situ*	20	0.7	0
NF5	2% CNF	*In situ*	20	0.7	0
CF1	1% CF10	*In situ*	2	2	0
CF2	1% CF10	Attached	2	2	0
CF3	1% CF10	*In situ*	2	2	0
CF4	1% CF10	*In situ*	2	0.5	7.5
CF5	1% CF10	*In situ*	2	0.5	15
CF6	1% CF10	*In situ*	2	0.5	22.5
CF7	1% CF10	Attached	2	2	7.5
CF8	1% CF10	Attached	2	2	15
CF9	1% CF10	Attached	2	2	22.5
CF10	1% CF10	*In situ*	2	2	7.5
CF11	1% CF10	*In situ*	2	2	15
CF12	1% CF10	*In situ*	2	2	22.5
CF13	1% CF10ox	*In situ*	2	2	30
CF14	1% CF10ox	*In situ*	2	0.5	30
CF15	1% CF10ox	*In situ*	2	2	30
CF16	1% CF10	*In situ*	2	2	30
CF17	1% CF10	*In situ*	2	0.5	30
CF18	1% CF10	Attached	2	2	30
CF19	1% CF3ox	*In situ*	2	2	30
CF20	1% CF3ox	*In situ*	2	0.5	30
CF21	1% CF3ox	*In situ*	2	2	30

**Figure 2:**
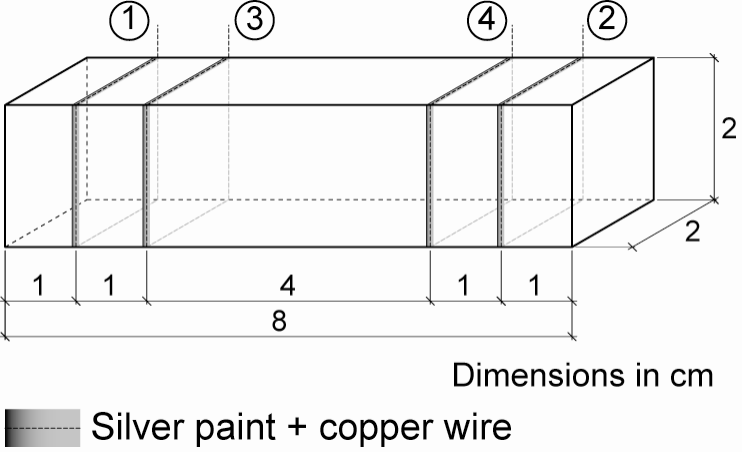
Electrical contacts: 1 and 2 are for current input; 3 and 4 for voltage measure.

In order to form four electrical contacts, as needed for the four-probe method, electrically conductive silver paint (Pelco Conductive Silver 187) was applied around the perimeter at four interior planes, which were parallel to the end surfaces (Figure 2). Afterwards, four copper wires were wrapped around each silver painted perimeter. 

### 2.2. Strain-Sensing and Damage-Sensing Test Setup

**Figure 3:**
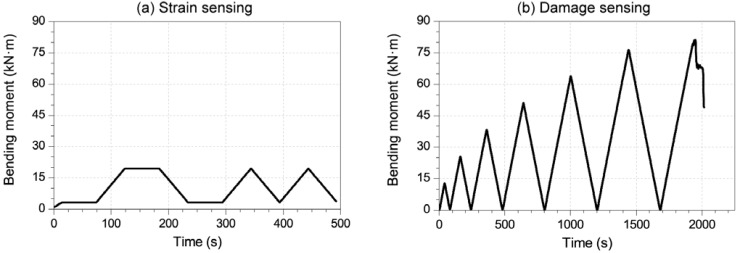
Loading configuration during: (**a**) strain-sensing tests; (**b**) damage-sensing tests.

Each sensing test was performed under four point bending, as shown in Figure 1a. All sensors were located between the two points where loading was applied; hence, the bending moment was the same for all of them. Figure 3 includes the bending moment for the middle cross section *versus* time for strain-sensing tests (Figure 3a) and damage-sensing tests (Figure 3b). The former presented loading and unloading cycles between 2 and 20 kN m, while the latter was made of cycles with progressively higher maximum loads, up to the beam’s failure.

For the electrical resistance measures during sensing tests (strain or damage), an electrical current intensity between the outer contacts was fixed at 1 mA with an AC/DC current source (model Keithley 6220) (Figure 2), while the voltage was measured between inner contacts using a digital multimeter (Keithley Model 2002). Hence, resistance may be calculated applying Ohm’s law. An electromechanical press with a loading cell with a maximum load of 200 kN was used for all tests. Longitudinal strain was permanently monitored with a Vishay P3 extensometer and strain gages located in the middle cross section (five gages equally distributed along depth) and four more gages on the upper face evenly distributed through the beam’s length (Figure 1c).

## 3. Results and Discussion

Before any multifunctional property was tested, the mechanical properties of the concrete used in the beam were measured. The average compressive strength and elastic modulus for cylindrical specimens tested according to European standards (UNE 83304:1984 and UNE 83316:1996 for strength and modulus) were 45.0 ± 0.3 MPa and 38851 ± 801 MPa, respectively. In the following discussion, several aspects have been analyzed, e.g., the type of conductive admixture (*i.e.*, CF or CNF), the influence of the sense of the applied stresses or strains (tension or compression) and the dimensions or location of the sensors. In a former analysis, all results of elastic (strain-sensing) tests are included, and later, the existence of a damage sensing mechanism in the same sensors is discussed. 

### 3.1. Strain-Sensing Tests’ Results

**Figure 4:**
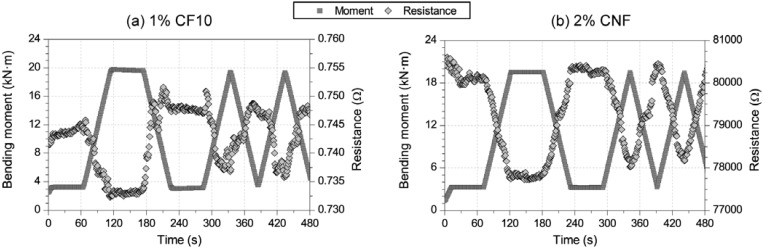
Electrical resistance and bending moment in the middle cross section *versus* time for sensors located on the compression side of the RC beam and with different conductive admixtures: (**a**) 1% CF10; (**b**) 2% CNF.

**Figure 5:**
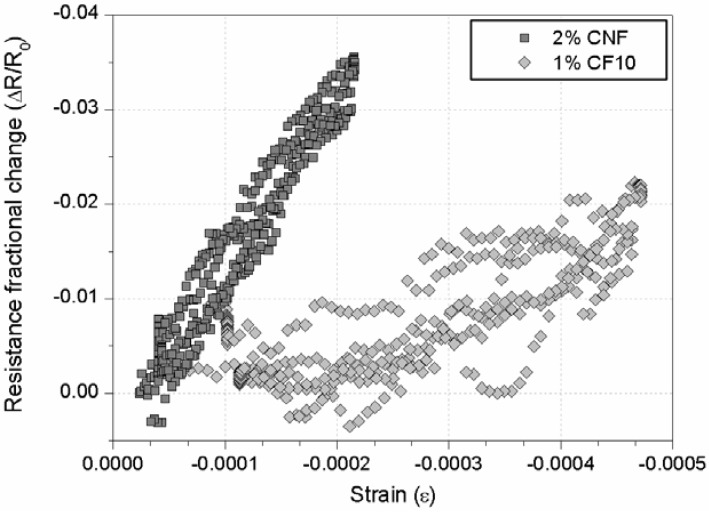
Electrical resistance *versus* longitudinal strain for sensors located on the compression side of the RC beam and with 1% CF10 or 2% CNF additions, respectively.

First of all, Figure 4 shows an example of strain-sensing capacity for both conductive admixtures (a) 1% CF10 and (b) 2% CNF. Each figure includes resistance and bending moment (in the section between loading points) data *versus* time for sensors located in the compression (upper) side of the beam. The electrical resistance of each composite is quite different, only 0.75 Ω for the long CF, whereas it increased up to approximately 2 kΩ for a CNF sensor of equal dimensions. These results are perfectly logical if compared to previous research [1,8,9,10]. For both cement pastes, the electrical response tends to follow the mechanical input suffered by each sensor, *i.e.*, lower electrical resistances for higher compressive strains. In order to make a comparison between those two additions, the gage factor (GF) according to Equation 1 should be calculated. This parameter can be clearly seen in a fractional change of resistance *versus* strain plot. Figure 5 includes this curve for these two sensors, and a higher sensitivity can be noted for the CNF sensors compared to the CF one.

**Figure 6:**
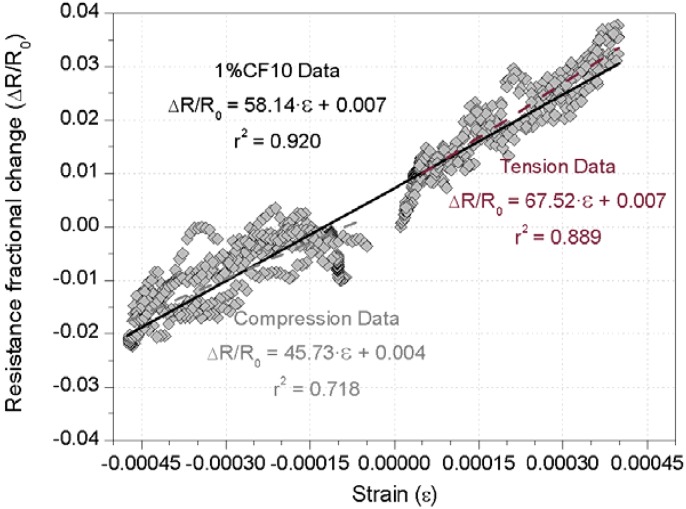
Comparison of fractional resistance-strain curves of 1% CF10 sensors under different strain conditions: compression (left curve) or tension (right curve).

A second variable of analysis was the influence of the composites location, *i.e.*, if the sensor was under tension or compression. For this particular purpose, in Figure 6, only the resistance/strain curves have been represented for a 1% CF10 cement paste; three linear regression functions have been included (two corresponding to each sensor considered separately and a third one for all data together). The electrical behavior upon compression has been previously discussed, and if tension is applied, hence, the electrical resistance is increased for higher strain levels, as previously reported [11,13,16,18]. The slope of each equation given in Figure 6 corresponds directly to the GF, *i.e.*, the sensors sensing capacity. If each sensor is analyzed independently, the tensioned one showed both higher GF and a better r^2^ Pearson coefficient than its compressed counterpart. However, if no distinction is made between sensors of the same dosage and dimensions, a very good linear adjustment is obtained, as it can be seen for the 0.92 r^2^ value of the third function.

**Figure 7:**
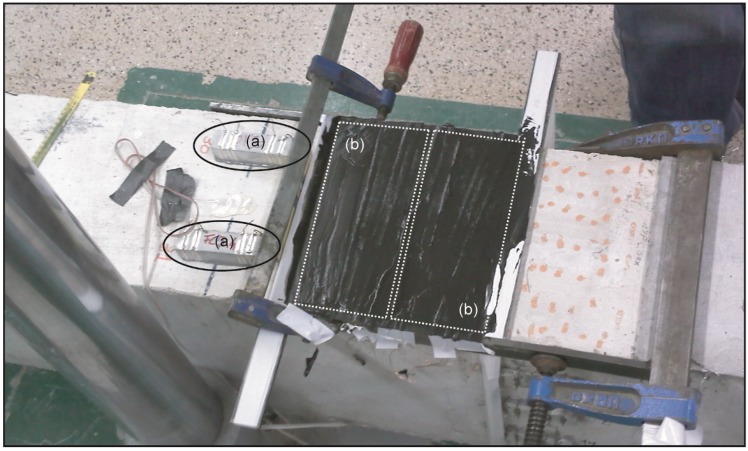
Class of sensor according to its dimensions: (**a**) 2 × 2 × 8 cm³; (**b**) 0.7 × 20 × 8 cm³.

The effect of the sensor’s dimensions has been the last variable of study for the strain sensing phenomena. For the CNFCC, two different sensor configurations were considered (Table 3), on one side, small samples (2 × 2 × 8 cm³), with the same ratios of uniaxial prior works [1,9,10], were attached to the beam, while, on the other hand, a continuous layer (0.7 × 20 × 8 cm³) less thick and as wide as the RC beam was poured directly upon the conventional concrete (type a and b respectively in Figure 7).

**Figure 8:**
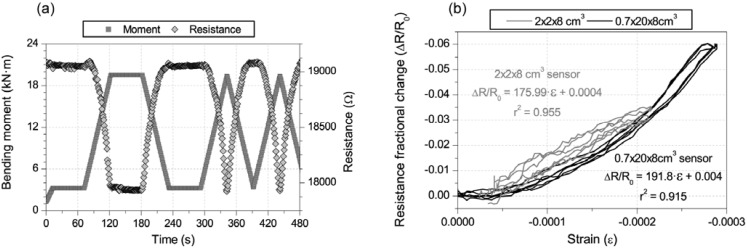
Comparison of 2% CNF (addition by cement mass) sensors: dimensions influence 2 × 2 × 8 cm³ *vs.* 0.7 × 20 × 8 cm³. (**a**) Electrical resistance and bending moment in the middle cross section *vs.* time for the 0.7 × 20 × 8 cm³ sensor; (**b**) resistance fractional change *vs.* longitudinal strain curves comparison between sensors NF2 and NF4.

Figure 8 includes results for 2% CNF cement pastes of each sensor type explained above and located in the compression side of the beam. Figure 8a shows the time evolution of bending moment and electrical resistance for the thin layer. The 2 × 2 × 8 cm³ counterpart has been previously discussed in Figure 4b. No difference can be seen in each graph between the loading-unloading cycles, and the constant stress part of the test, *i.e.*, for the same loading conditions, the same change in the resistance, was registered regardless of how the load was applied or how much time it lasted. In Figure 8b, the equivalent resistance fractional change-strain curves are plotted. Both linear correlations present r² coefficients higher than 0.91, but the GF of the continuous layer was a little higher (191.8) than the smaller sensor (175.99). This higher sensitivity may be due to a better strain transmission between the paste and concrete, improved by the lower layer’s thickness, which enables a lower strain gradient, as reported in authors’ previous research [9,10]. Moreover, the second curve also points to some second degree effects that should be considered to define more accurately the sensing process, e.g., for the curve in Figure 8b, a second degree polynomial would be a better fit function with a r² coefficient of 0.997 [9,10].

**Figure 9:**
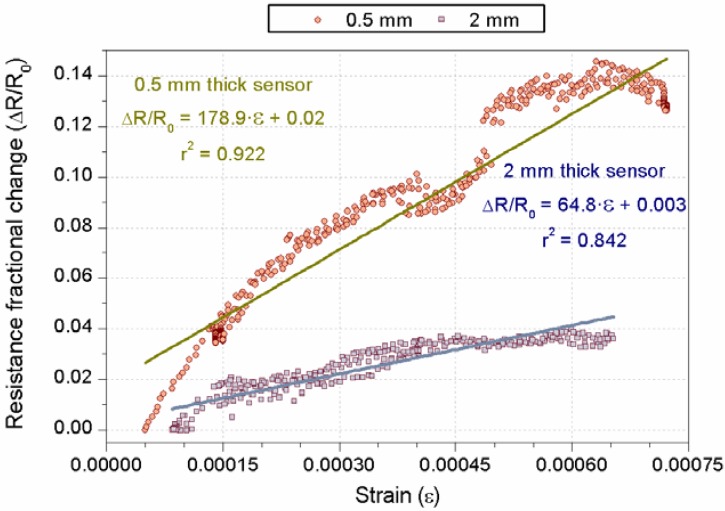
Comparison of 1% CF10 sensors with different thicknesses (0.5 mm and 2 mm).

Figure 9 includes another example of the self-sensing capacity improvement, which can be achieved by a smaller thickness of the sensor, but using CF as a conductive admixture this time. Two resistance fractional change-strain curves have been registered for sensors CF9 and CF14 (see Table 3); both were located in a tensioned part of the RC beam and were 2 mm and 0.5 mm thick, respectively. The GF calculated for the thinner sensor was almost thrice than CF9 sensor. This sensitivity enhancement could also have been boosted by a better anchorage of fibers in the matrix, due to the oxidation process of CF, which were used in CF14 [31].

Finally, to sum up this first stage of the report, both CF and CNF cement pastes were capable of acting as a strain sensor attached to an ordinary RC beam under flexure. Moreover, CNF composites were shown to be a more sensitive sensor, as indicated by their higher gage factor, up to 191.8. 

### 3.2. Damage-Sensing Results

**Figure 10:**
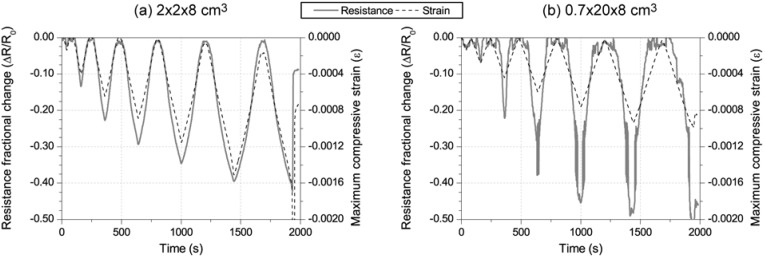
Damage sensing test for sensors NF2 (**a**) and NF4 (**b**), Resistance fractional change and longitudinal strain at sensor’s location are represented, both *vs.* time.

After all the reversible tests had been done, the damage sensing test was carried out. As the CNF composites had shown better results as strain sensors, the decision to measure them exclusively was taken. Hence, the second loading scheme explained in Figure 3b was applied, and the electrical resistance was registered for sensors NF2 and NF4 (Table 3). Figure 10 includes the curves corresponding to mechanical and electrical properties of both CNF sensors, up to the beam’s failure. If a damage sensing mechanism were triggered, an irreversible response should be observed in the composite’s electrical resistance [9,10,11]. Hence, the initial resistance, once the load had been removed, should have changed. In Figure 10a, corresponding to a 2 × 2 × 8 cm³ CNF sensor, the strain curve presents certain irreversible values, progressively higher, since the fourth cycle (≈800 s). At the same time, the resistance change almost stays reversible always. A little decrease in initial resistivity can be seen for higher stresses, but not related in any case to damage sensing phenomena. 

**Figure 11:**
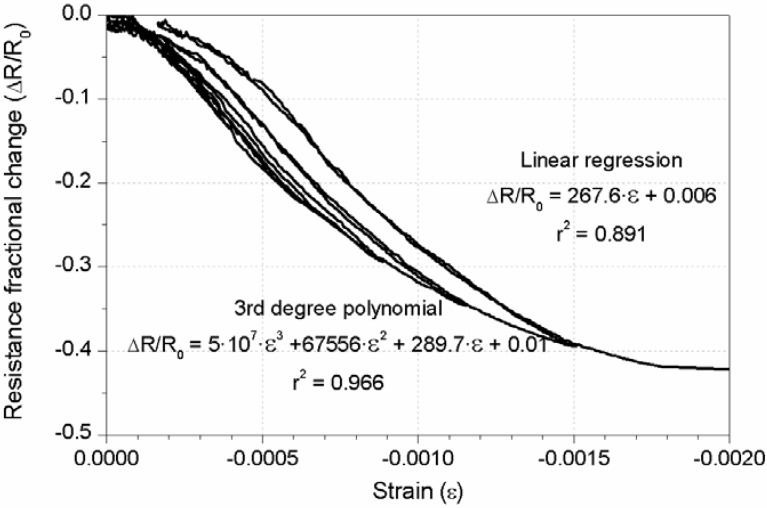
Resistance fractional change *vs.* strain for sensor NF2 damage sensing test.

A different behavior was registered in the thinner plate (sensor NF4) and has been plotted in Figure 10b. This time, even for a lower strain level, some degradation of the electrical properties was detected, *i.e.*, the strain sensing capacity, as explained above, was totally lost (for strains lower than 300 µε) and higher resistance decreases were observed for each cycle’s maximum load. As an initial conclusion for damage tests, no CNF composite was capable of detecting the RC beam’s damage. Nonetheless, another possible application of these materials can be concluded if further analysis of NF2 data is made. Figure 11 shows the resistance change-strain curve for all the failure test cycles. However, no apparent damage sensing mechanism can be seen, the resistivity-strain relationship characteristic of strain sensing phenomena is guaranteed for almost the entire process. Only the last cycle showed some permanent initial resistance change, when the composite reached 2000 µε. Similar results have been previously reported for CNF samples under uniaxial compression [10]. Hence, a CNF cement paste was capable of acting as a strain sensor attached to a RC beam, even if the structure was close to collapse, but it was not applicable as a damage sensor, due to the low strains that concrete admits.

## 4. Conclusions 

Multiple sensors based on multifunctional cement composites (with the addition of carbon fibers and carbon nanofibers) were attached to a conventional reinforced concrete beam. After the experimental program, which included both strain- and damage-sensing tests, the following conclusions can be drawn.

Both admixtures, CNF and CF, were suitable as conductive admixture to fabricate cement composites capable of measuring strains on the surface of a structural element, regardless if the local stresses were tension or compression. Furthermore, almost no differences were found between gage factors calculated for 1% CF composites (with equal dimensions) located on the upper and lower sides of the same beam’s section. A cement paste with an addition of 2% CNF (by mass of cement) was the most sensitive dosage, with calculated gage factors around 190. The thinner the sensor was, the higher sensing capacity the composite had.

CNF cement composites were unable to develop any damage-sensing mechanism when attached to a RC element, because of the magnitude of strains, which caused the concrete’s failure. However, they were capable of strain sensing during all the collapse process.

## References

[1] Baeza F.J., Zornoza E., Andión L.G., Ivorra S., Garcés P. (2011). Variables affecting strain sensing function in cementitious composites with carbon fibers. Comput. Concr..

[2] del Moral B., Galao O., Antón C., Climent M.A., Garcés P. (2012). Usability of cement paste containing carbon nanofibers as an anode in electrochemical chloride extraction from concrete. Mater. Constr..

[3] Garcés P., Carmona J., Galao O., Zornoza E., Climent M.A. Carbon Nanofibre Cement Paste as Anode for Electrochemical Chloride Removal. NICOM4 Nanotechnology in Construction, Proceedings of the 4th International Symposium on Nanotechnology in Construction.

[4] Pérez A., Climent M.A., Garcés P. (2010). Electrochemical extraction of chlorides from reinforced concrete using a conductive cement paste as an anode. Corros. Sci..

[5] Zornoza E., Galao O., Baeza F.J., Garcés P. Electromagnetic Interference Shielding of Cement Pastes with Carbon Nanofibres. NICOM4 Nanotechnology in Construction, Proceedings of the 4th International Symposium on Nanotechnology in Construction.

[6] Zornoza E., Catalá G., Jiménez F., Andión L.G., Garcés P. (2010). Electromagnetic interference shielding with Portland cement paste containing carbon materials and processed fly ash. Mater. Constr..

[7] Wu J., Chung D.D.L. (2005). Pastes for electromagnetic interference shielding. J. Electron. Mater..

[8] Baeza F.J., Chung D.D.L., Zornoza E., Andión L.G., Garcés P. (2010). Triple percolation in concrete reinforced with carbon fiber. ACI Mater. J..

[9] Baeza F.J. (2011). Función de percepción de la deformación en matrices cementicias conductoras mediante adición de fibras de carbono. Ph.D. Thesis.

[10] Galao O. (2012). Matrices cementicias multifuncionales mediante adición de nanofibras de carbono. Ph.D. Thesis.

[11] Chung D.D.L. (2003). Multifunctional Cement-Based Materials.

[12] Chen P.W., Chung D.D.L. (1993). Carbon fiber reinforced concrete as a smart material capable of non-destructive flaw detection. Smart Mater. Struct..

[13] Chen P.W., Chung D.D.L. (1996). Concrete as a new strain/stress sensor. Compos. B Eng..

[14] Chung D.D.L. (2001). Cement-matrix composites for thermal engineering. Appl. Therm. Eng..

[15] Galao O., Gomis J., Zornoza E., Baeza F.J., Garcés P. Heating Function of Carbon Nanofibre Cement Pastes. NICOM4 Nanotechnology in Construction, Proceedings of the 4th International Symposium on Nanotechnology in Construction.

[16] Chung D.D.L. (2000). Cement-matrix composites for smart structures. Smart Mater. Struct..

[17] Chung D.D.L. (2001). Functional properties of cement-matrix composites. J. Mater. Sci..

[18] Reza F., Batson G.B., Yamamuro J.A., Lee J.S. (2003). Resistance changes during compression of carbon fiber cement composites. J. Mater. Civil Eng..

[19] Chung D.D.L. (2003). Damage in cement-based materials, studied by electrical resistance measurement. Mater. Sci. Eng. R..

[20] Hou J., Chung D.D.L. (1997). Cathodic protection of steel reinforced concrete facilitated by using carbon fiber reinforced mortar or concrete. Cem. Concr. Res..

[21] Bertolini L., Bolzoni F., Pastore T., Pedeferri P. (2000). Effectiveness of a conductive cementitious mortar anode for cathodic protection of steel in concrete. Cem. Concr. Res..

[22] Tuan C.Y., Yehia S. (2004). Evaluation of electrically conductive concrete containing carbon products for deicing. ACI Mater. J..

[23] Li G.Y., Wang P.M., Zhao X. (2007). Pressure-sensitive properties and microstructure of carbon nanotube reinforced cement composites. Cem. Concr. Compos..

[24] Yu X., Kwon E. (2009). A carbon nanotube/cement composite with piezoresistive properties. Smart Mater. Struct..

[25] Han B.G., Yu X., Kwon E. (2009). A self-sensing carbon nanotube/cement composite for traffic monitoring. Nanotechnology.

[26] Saafi M. (2009). Wireless and embedded carbon nanotube networks for damage detection in concrete structures. Nanotechnology.

[27] Howser R.N., Dhonde H.B., Mo Y.L. (2011). Self-sensing of carbon nanofiber concrete columns subjected to reversed cyclic loading. Smart Mater. Struct..

[28] Galao O., Zornoza E., Baeza F.J., Bernabeu A., Garcés P. (2012). Effect of carbon nanofiber addition in the mechanical properties and durability of cementitious materials. Mater. Constr..

[29] Alcaide J., Alcocel E., Puertas F., Lapuente R., Garcés P. (2007). Carbon fibre-reinforced, alkali-activated slag mortars. Mater. Constr..

[30] Ivorra S., Garcés P., Catalá G., Andión L.G., Zornoza E. (2010). Effect of silica fume particle size on mechanical properties of short carbon fiber reinforced concrete. Mater. Design.

[31] Catalá G., Ramos-Fernández E.V., Zornoza E., Andión L.G., Garcés P. (2011). Influence of the oxidation process of carbon materials on the mechanical properties of cement mortars. J. Mater. Civil Eng..

[32] Garcés P., Fraile J., Vilaplana-Ortego E., Cazorla D., Alcocel E.G., Andión L.G. (2005). Effect of carbon fibers on the mechanical properties and corrosion levels of reinforced Portland cement mortars. Cem. Concr. Res..

[33] Garcés P., Zornoza E., Alcocel E.G., Galao O., Andión L.G. (2012). Mechanical properties and corrosion of CAC mortars with carbon fibers. Constr. Build. Mater..

[34] Garcés P., Andión L.G., Varga I., Catalá G., Zornoza E. (2007). Corrosion of steel reinforcement in structural concrete with carbon material addition. Corros. Sci..

[35] Chung D.D.L. (2004). Cement-matrix structural nanocomposites. Met. Mater. Int..

[36] Sanchez F., Ince C. (2009). Microstructure and macroscopic properties of hybrid carbon nanofiber/silica fume cement composites. Compos. Sci. Technol..

[37] Tyson B.M., Abu Al-Rub R.K., Yazdanbakhsh A., Grasley Z. (2011). Carbon nanotubes and carbon nanofibers for enhancing the mechanical properties of nanocomposite cementitious materials. J. Mater. Civil Eng..

[38] Bortz D.R., Merino C., Martin-Gullon I. (2011). Carbon nanofibers enhance the fracture toughness and fatigue performance of a structural epoxy system. Compos. Sci. Technol..

[39] Soroushian P., Nagi M., Okwegbu A. (1992). Freeze-thaw durability of lightweight carbon-fiber reinforced cement composite. ACI Mater. J..

